# Busulfan Administration Flexibility Increases the Applicability of Scid Repopulating Cell Assay in NSG Mouse Model

**DOI:** 10.1371/journal.pone.0074361

**Published:** 2013-09-17

**Authors:** Jean Chevaleyre, Pascale Duchez, Laura Rodriguez, Marija Vlaski, Arnaud Villacreces, Véronique Conrad-Lapostolle, Vincent Praloran, Zoran Ivanovic, Philippe Brunet de la Grange

**Affiliations:** 1 Etablissement Français du Sang – Aquitaine Limousin (EFS-AqLi), Bordeaux, France; 2 CNRS (UMR 5164), Bordeaux, France; 3 Université Bordeaux Segalen (UMR 5164), Bordeaux, France; Wake Forest Institute for Regenerative Medicine, United States of America

## Abstract

**Background:**

Xenotransplantation models allowing the identification and quantification of human Hematopoietic stem cells (HSC) in immunodeficient mice remain the only way to appropriately address human HSC function despite the recent progress in phenotypic characterization. However, these *in* *vivo* experiments are technically demanding, time consuming and expensive. Indeed, HSCs engraftment in mouse requires pre-conditioning of animals either by irradiation or cytotoxic drugs to allow homing of injected cells in specific stem cell niches and their subsequent expansion and differentiation in bone marrow. Recently, the development of busulfan pre-conditioning of animals improved the flexibility of experimentation in comparison with irradiation.

**Design and Methods:**

In order to further facilitate the organization of these complex experiments we investigated the effect of extending the period between mice pre-conditioning and cell injection on the engraftment efficiency. In the meantime, we also explored the role of busulfan doses, mouse gender and intravenous injection route (caudal or retro orbital) on engraftment efficiency.

**Results and Conclusion:**

We showed that a period of up to 7 days did not modify engraftment efficiency of human HSCs in NSG model. Moreover, retro orbital cell injection to female mice pre-conditioned with 2x25 mg/kg of busulfan seems to be the best adapted schema to detect the human HSC in xenotransplantation experiments.

## Introduction

Hematopoietic stem cells (HSC) cannot be distinguished from Hematopoietic Progenitors (HP) by phenotypic and/or molecular analysis. Indeed, even though human CD34^+^CD38^-/low^CD90^+^CD45RA^-^ hematopoietic cell population [1] is very enriched in stem cells, it still contains a non-negligible proportion of HP. *In vivo* xenotransplantation assays are thus mandatory to reveal and quantify human HSC. This test is based on the unique functional capacity of HSC to durably engraft recipients in which they self-renew, expand and give rise to a progeny of more mature hematopoietic cells. To address human HSC, CB17-SCID and NIH Beige-Nude-Xid mice strains were used up to 1995. Afterwards, NOD/SCID model, exhibiting a more severe immune deficit due to the combined alteration of NK cells, complement and macrophages, was used thereafter. The recently developed NOG (NOD/Shi-*scid*/IL-2Rγ^null^) and NSG (NOD.Cg-*Prkdc* ^*scid*^ *Il2rg tm1Wjl*/SzJ) mouse strains are now considered as the most efficient model for studying human HSC [2-6], commonly named “Scid Repopulating Cells” (SRC).

The immuno-deficient models are able to evidence the functional heterogeneity of HSC population that contains short term (ST-) and long term (LT-) SRC. These functional SRC subpopulations are distinguished by the period between cell injection and analysis of their progeny (6-8 weeks and 12-14 weeks for ST- and LT-SRC, respectively) or by using serial transplantations (limited primary or serial (secondary and more) engraftment capacity for ST- and LT-SRC, respectively). This xenotransplantation procedure, based on the functional characteristic and definition of HSC, is presently the gold standard to study these cells. Successful transplantation of HSC requires a partial or total suppression of the recipient hematopoiesis. One of the critical point is represented by the pre-conditioning of the animals before they receive infusion of cell suspension. Indeed, it facilitates the homing of HSC in specific BM niches and their later self-renewal, proliferation and differentiation inside the whole bone marrow space by a combined suppression of endogenous bone marrow hematopoiesis and stimulation of a major feedback hematopoietic regeneration process. Despite the fact that it is tedious to organize, sub-lethal or lethal X or Gamma rays irradiation is the most common conditioning regimen. Alternatively, some authors proposed recently to use chemotherapeutic agents such as busulfan and 5-fluorouracil that induce similar hematopoietic effects [7,8] while being easier to set-up and less expensive than irradiation.

Despite the use of the recently developed NOG (NOD/Shi-*scid*/IL-2Rγ^null^) and NSG (NOD.Cg-*Prkdc* ^*scid*^ *Il2rg tm1Wjl*/SzJ) immunodeficient mouse strains that lowered the engraftment threshold [2,6], the use of non standardized procedures leads to frequent inter-laboratories variations. Based on the recent conditioning regimen that proposes two i.p. injections of busulfan 48 and 24 hours before IV injection of HSC [8], we tried to reduce the time schedule constraints of this procedure and to delineate in the meantime the best procedure to address human cord blood CD34+ HSC. For this purpose we compared the NSG engraftment by using the classical procedure or modifications targeting busulfan dose, gender of animals, injection route, and the period between NSG mouse conditioning and cell injection. Among the results we obtained, we showed most interestingly that prolonging the period up to 7 days between conditioning busulfan regimen (25mg/Kg (x2)) and cells injection did not decrease the HSC engraftment.

## Design and Methods

### Ethic statement

The informative consent to use CB for experimental purposes (in case it would not suitable for banking) was systematically obtained from mothers before sampling. The experiments were performed on discarded CB units in compliance with French regulation (article R1243-49 of “*Code de Santé Publique*”) and with a related authorization (N° AC 2008-749) granted to the Laboratory and concerning activity of conservation and preparation of human origin samples for scientific purposes. The experiments presented in this paper are authorized by Ethical Committee of Bordeaux (authorization N° 50120213-A) in the context of a project of our group. All animal experiments were performed in agreement with the French regulation (licenses granted to PBG and ZI N° A33 12 054 and N° B33 11 005, respectively).

All participants provided written informed consent.

### CD34^+^ Cord Blood cells isolation

CD34^+^ cells were isolated from cord blood (CB) samples rejected from the EFS (Etablissement Français du Sang) CB bank because of a volume or CD34^+^ cell number lower than the banking thresholds. CD34^+^ cells were purified using MACS technology according to the manufacturer instructions (Miltenyi Biotech, Paris, France). The selection efficiency (>95% CD34^+^ cells in all samples) was assessed by flow cytometry (FACS Canto II, Becton Dickinson) by labeling an aliquot of cell suspension with an anti CD34 antibody (Becton Dickinson, Rungis, France). Freshly isolated CD34^+^ cells were either directly injected into mice or frozen in fetal bovine serum 10% DMSO for later use.

### Pre-conditioning of mice with busulfan

According to the previously established protocol [8], six to 10-week-old female and male NSG mice (from A2 central animal-keeping facility of University of Bordeaux Segalen) were conditioned by 2 intraperitoneal injections of 20, 25 or 30 mg/kg Busulfan (Busilvex^®^, Pierre Fabre, Boulogne, France) at 24 hours interval. The individual dose of busulfan was adjusted according to mice weight.

### Scid-Repopulating Cells (SRC) Assay

#### Cells injection

One, two or seven days after the second busulfan injection, 100, 500 or 1000 human CD34^+^ cells (re-suspended in 200µL of albumin 4% (LFB, France) were i.v. injected either in the caudal vein or retro orbital sinus. Positive controls (mice injected with 15000-20000 CD34^+^ cells) and negative controls (non-injected mice) were included in each experiment. Mice were placed in a restraint cage for caudal vein injection and anesthetized using Isoflurane gas mixture (2.5%) for injections in retro orbital sinus. Animals were daily followed for their social behaviour, fur quality, dramatic weight loss (>20% of total body weight) and were euthanized when necessary by competent operator using cervical dislocation method.

#### Human cell engraftment analysis

8 weeks after CD34^+^ cell injections, the animals were euthanized by cervical dislocation, their femora isolated and the BM flushed with 1 ml of RPMI 1640 complemented with human albumin 4%. Cells were counted and an aliquot incubated with a mixture of FITC-coupled anti-human CD45, PE-coupled anti-human CD19 and APC-coupled anti-human CD33 antibodies for 20 minutes at 4°C in the dark. Washed cells were analyzed on a FACSCalibur or FACS CANTO II (both from Becton Dickinson) to detect and quantify the level of human cells engraftment.

To detect the committed progenitors (Colony Forming Units - CFUs) of human origin in murine bone marrow, 15 and 30 µl of femoral cell suspension (see above) were seeded in duplicate in 250 µl of semi solid (methylcellulose) culture medium (Stem Alpha-1; Stem Alpha SA) supplemented with 10% fresh frozen human plasma AB (EFS Aquitaine-Limousin, Bordeaux, France), 25 ng/mL (rHu IL-3) (Pepro Tech, London), 25 ng/mL rHu GM-CSF) (R&D Systems, Europe, Lille, France), 50 ng/mL rHu SCF (Amgen-Roche, Neupogen) and 3 U/mL rHu Erythropoietin (EPO) (Tebu- Bio, Le Perray, France) [9]. After 14 days of incubation at 37°C, 5% CO_2_, human colonies (>50 cells) were enumerated using an inverted microscope. The discrimination between CFUs from human and mouse origin is based on the development of colonies composed of hemoglobinized cells (red color) for BFU-E (the murine-origin BFU-E do not grow in the conditions used notably due to the absence of specific murine IL-3); the development of colonies with normal shape and composed of big clearly round cells, while the mouse progenitors in these conditions develop only some clusters of bad-shape cells for CFU-GM. A control culture with only mouse cells seeded in methyl cellulose is included in each experiment.

### Statistical analysis

All comparisons were assessed using Mann-Whitney Test. Result were considered significant if *P* value was less than 0.05. *P <0.05; ** *P* <0.005; *** *P* <0.0005.

## Results

### Busulfan conditioning dosage interferes with weight, mortality and engraftment level of NSG mice (fig 1)

**Figure 1:**
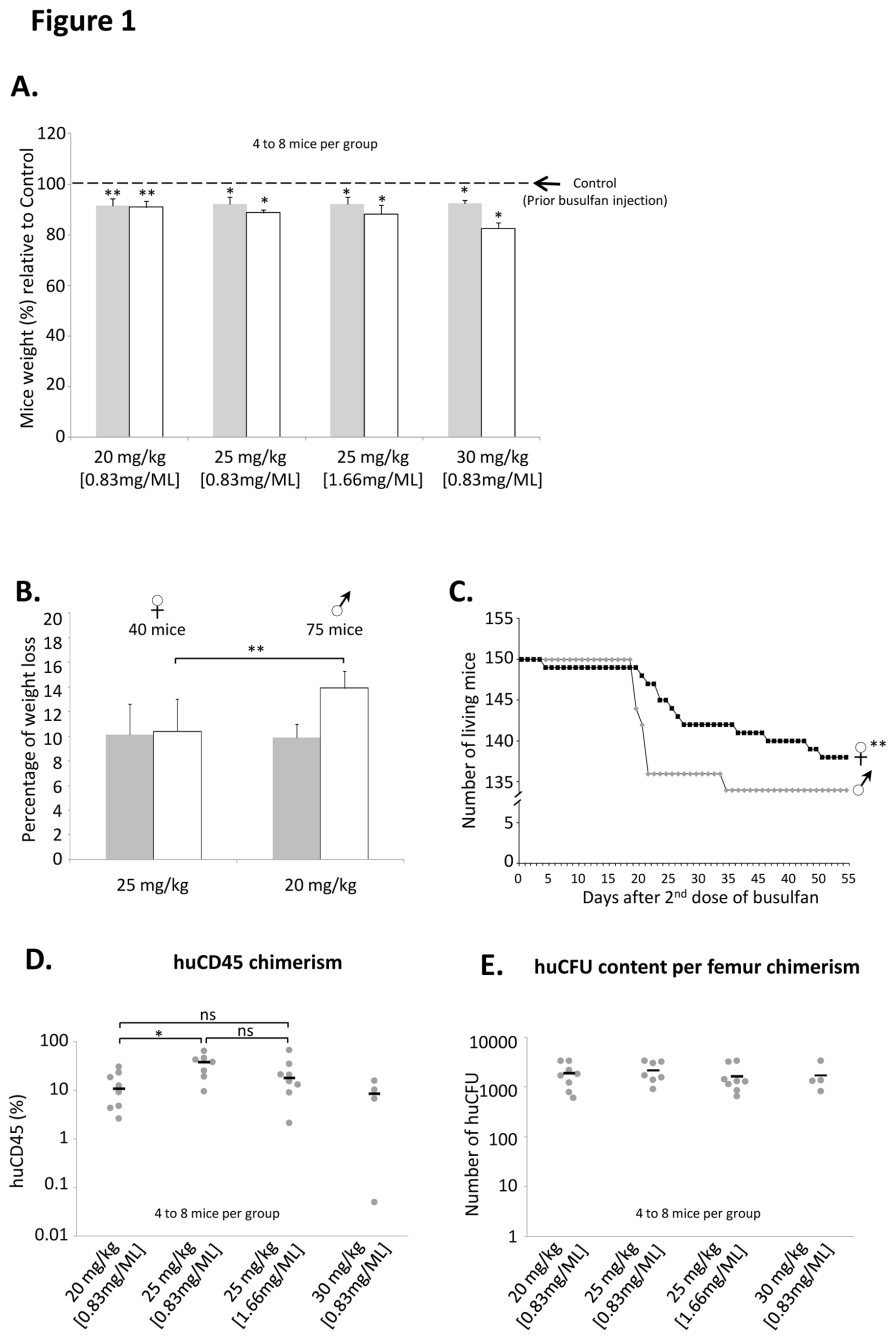
Evaluation of busulfan pre-conditioning in mice. Mice received increasing doses of busulfan (2 injections) and were weighted prior and after 24 hours following each injection. **A.** Percentage (%) of weight relative to weight prior injection (---) and measured 24 hours after the first (▪) and the second (□) injection of different doses of busulfan. **B.** Percentage of weight loss according to gender 24 hours after the first (▪) and the second (□) injection. **C.** Survival of mice according to gender. **D-E.** Mice received 1000 non manipulated CD34+ cells by i.v. caudal injection and were analyzed 8 weeks later for huCD45 chimerism (D) and huCFU content (E) in their bone marrow. Each mouse represented by (●), Median represented by (─).

Intra peritoneal injections of busulfan were performed 48h and 24h before cell injection with 3 different doses (20, 25 and 30mg/kg; diluted at 0.83 mg/mL). Concerning the 25mg/kg dose we also tested a 2 fold more concentrated busulfan solution, i.e. 1.66 mg/mL in order to decrease the injected volume without losing efficiency or increasing toxicity. As shown in Figure 1A, the weight of all mice (8 mice for 20mg/kg, 7 mice for 25mg/kg and for 30mg/kg and 8 mice for 25mg/kg using busulfan at 1.66mg/mL) decreased significantly 24 hours after the 1^st^ injection of busulfan whatever the dose used. In addition, 24 hours after the 2^nd^ injection the weight loss still increased in parallel with the dose of busulfan delivered (9.4% for 20mg/kg (8 mice), 11.2% for 25mg/kg (6 mice) and 16% for 30mg/kg (4 mice)). Males are more sensitive than females to IP Busulfan since their weight loss was more pronounced even for a lower dose of 20 mg/kg in males compared to 25mg/kg in females (up to 13.9% *vs* 10.4% for males and females respectively) (Figure 1B). This higher sensitivity of males is supported by the fact that the mortality rate of all injected males (whatever the dose of Busulfan) was higher than that of females starting from the third week after cell injection (10.7% and 8% respectively, n=56 mice per group, p=0.0048) (Figure 1C). With respect to engraftment efficiency, human CD45 BM chimerism was significantly higher 6 to 8 weeks after cell injection in mice conditioned with 25mg/kg (x2) of busulfan than in those conditioned with 20mg/kg (x2) (median 37.6±6.9% of huCD45 cells *vs* 10.7 ±3.5%, p=0.0089; Figure 1D). The use of a more concentrated busulfan solution (1.66 mg/mL instead of 0.83mg/mL) did not significantly change the engraftment level. Due to the high mortality induced by 30mg/kg (X2) busulfan we cannot conclude on its impact on the engraftment level. In contrast with its effect on the human CD45 chimerism, increasing doses of busulfan did not significantly change the number of human CFC cells (related to more primitive cells, mainly CFU-GM colonies ≤ 100 cells) contained in the mice bone marrow (Figure 1E).

### Engraftement Efficiency Is Not Modified by Increasing the Period from 1 to 7 Days between Busulfan Injection and Hematopoietic Cells Infusion (Fig 2)

**Figure 2:**
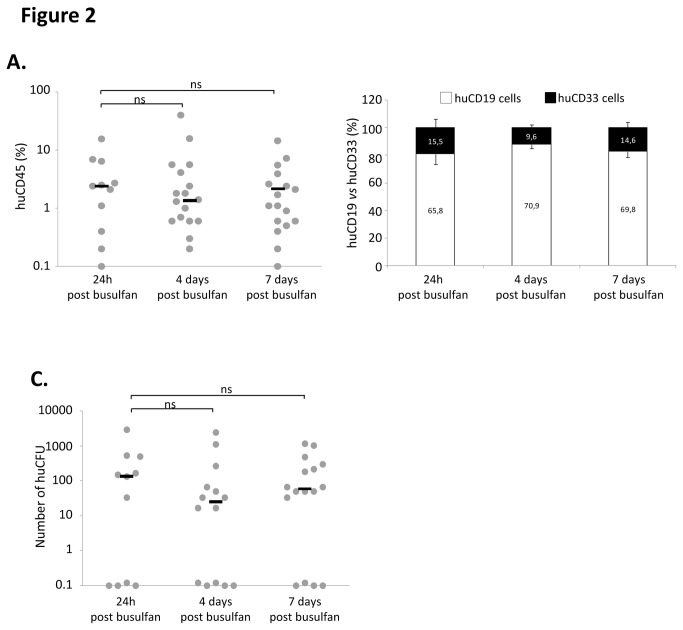
Impact on engraftment level of time delay between mice pre-conditioning and cell injections. Female mice received 2 doses of busulfan (25mg/Kg each) at 24 hours, 4 days or 7 days intervals. Mice received 1000 non manipulated CD34+ cells by caudal injection and their bone marrow were analyzed 8 weeks later for **A.** total huCD45 chimerism, **B.** B lymphoid (▪) versus myeloid (□) cells ration in CD45+ cells, and **C.** huCFUs content per femur. Each mouse represented by (●), Median represented by (─).

The common time-schedule of Busulfan conditioning is that cell injection is done 24 hours after the second dose. It is a significant technical constraint if one considers uncertainties concerning the regular availability of donor cells together with their frequently required pre-transplantation processing (cell sorting, cultures, cell transfections or transductions, etc) that complicates organization of *in vivo* experiments. In order to improve the flexibility of such studies, we compared the engraftment efficiency of cells injected 24 hours, 4 and 7 days after the second injection of busulfan. As shown in Figure 2A, despite lower engraftment level than in previous experiment (probably due to inter-individual variations in cord blood sample), the percentage of huCD45 cells 8 weeks after transplantation remained similar whatever the period tested (non-significant difference between median values: 2.4 ±1.4%, 1.4±1.8% and 2.2±0.6% at 24 hours, 4 days and 7 days, respectively; 11 to 17 mice cohorts; all mice received CD34^+^ cells from the same cord blood sample). Moreover, the expected B lymphoid *versus* myeloid ratio was not affected (Figure 2B). Finally, the number of huCFUs per mouse femur also remained similar (Figure 2C).

### Engraftment efficiency is slightly dependent on the intra venous injection route (fig 3)

The CB CD34^+^ cells (same number, same sample and same volume of medium (0.2 ml)) were injected 24h after the 2^nd^ busulfan injection either in the caudal vein or in the retro-orbital sinus of mice conditioned with 2x25 mg/kg of busulfan (0.83 mg/ml). The level of human cell engraftment was not significantly different between the two injection routes despite a 2.3 fold higher median percentage of huCD45 positive cells for retro orbital sinus injection (16 ±2.94%, non significant) compared to caudal vein injection (7 ±2.9%). However, the median number of CFUs per femur was significantly higher (3.1 times) when cells where injected in the retro orbital sinus suggesting a better efficiency of this injection route. Furthermore, whereas retro-orbital sinus injection resulted in positive engraftment of all tested mice, 2 (12.5%) were negative after caudal injection.

### The human engraftment threshold in NSG mice is sex-related (fig 4)

Due to the above described results, the following experiments were done using the retro orbital injection route. We tested here lower doses of 500 and 100 cells, in order to compare the minimal number of injected cells that produced a reliable engraftment in male and female NSG mice. In spite of very low median, huCD45 engraftment in both female and male injected with 100 CD34^+^ cells (0.4 ±0.62% and 0.2±0.07% respectively) showed a significant difference. Indeed, 13 out of the 14 females (93%) were beyond the positivity threshold (0.2%; defined on the basis of non-injected mice) with values up to 8.6% whereas only 6 out of 11 males (54.5%) were beyond this threshold with a maximal value of 0.8%. As expected, the huCD45^+^ cells chimerism increased with the number of cells injected in males and females. However, the cell dose dependent increase in human chimerism was higher in females than in males. Thus, engraftment increased by more than 4 fold in females when the number of cells injected increased from 500 to 1000 while it did not change in males. This led to a very strong discrepancy for the 1000 cells dose for which the proportion of huCD45^+^ cells is 12 fold higher in females than in males (12.7±7.88% and 1±2.31% respectively; p = 0.0087). Similar differences were also observed for the number of CFU per femur, but the significance between females and males was reached only for the highest cell dose (Figure 4B).

## Discussion

**Figure 3:**
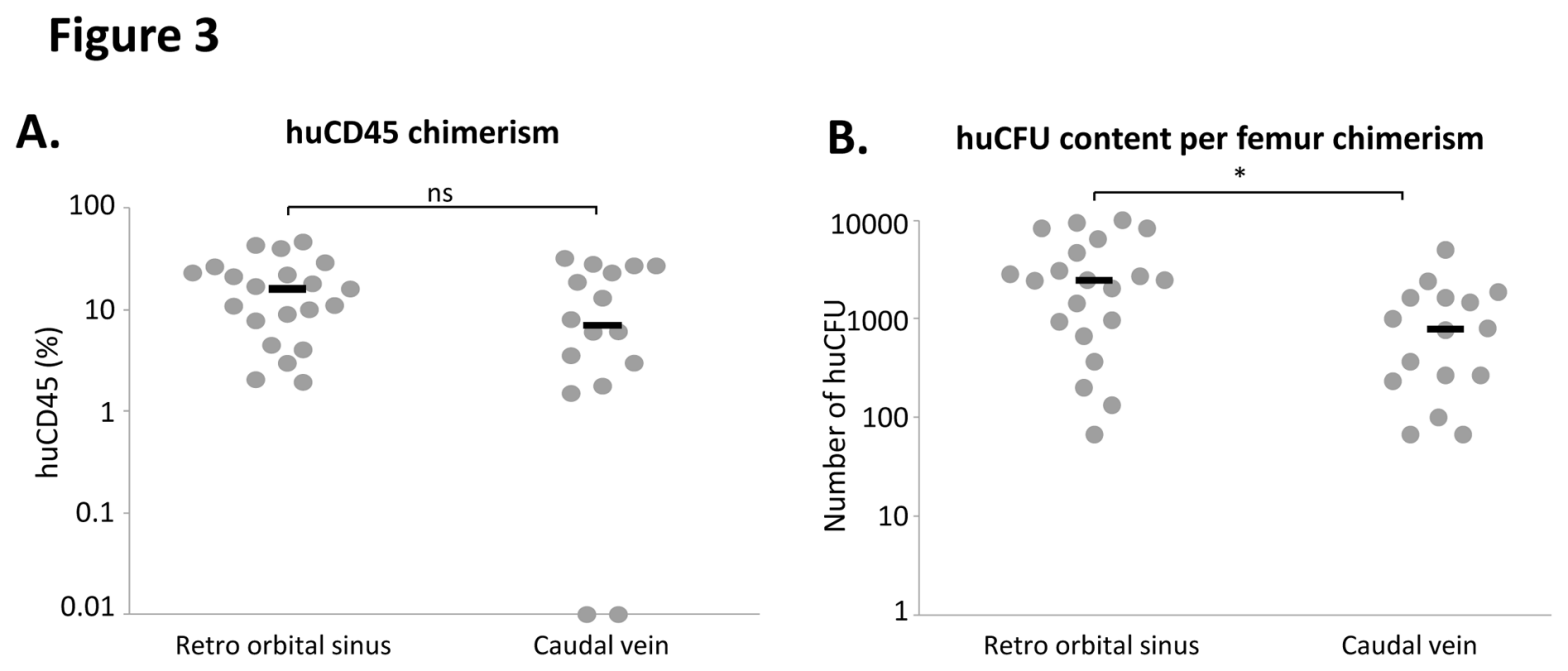
Comparison of caudal vein and retro orbital injections. After classical busulfan pre-conditioning (2x25 mg/Kg), female mice received 1000 non manipulated CD34+ cells by i.v. injection either in the caudal vein or retro orbital sinus. Their bone marrow were analyzed 8 weeks later for **A.** total huCD45 chimerism, **B.** huCFU content. Each mouse represented by (●), Median represented by (─).

**Figure 4:**
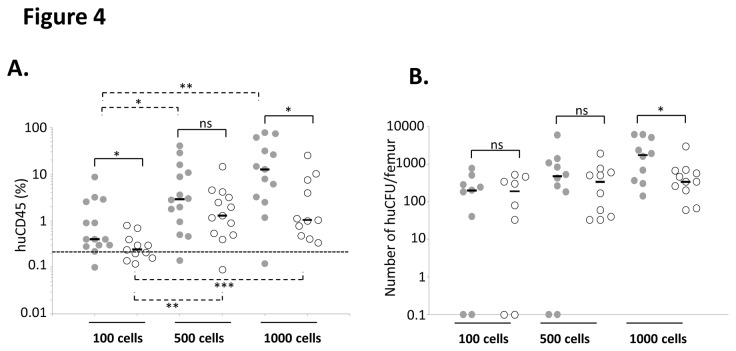
Impact of gender on human cells engraftment. After classical busulfan pre-conditioning (2x25 mg/Kg), female and male mice received 100, 500 or 1000 non manipulated CD34+ cells by i.v. injection in the retro orbital sinus. Their bone marrow were analyzed 8 weeks later for **A.** total huCD45 chimerism, **B.** huCFU content. Each mouse represented by ● (female) or ○ (male), Median represented by (─).

The Immuno-deficient NSG mouse strain is now widely used for the *in vivo* detection and quantification of human hematopoietic stem cells since it allows an enhanced engraftment compared to the “classical” NOD/SCID mice xenotransplantation model [2-6]. The first step of the procedure is to “condition” the receiver before the injection of human cells in order to ensure the best conditions for supporting the BM homing and development of HSC. Total body non-lethal irradiation of mice with x- or γ-rays, the most common conditioning regimen triggers several mechanisms, including the secretion of stem cell factor (SCF) which is critical for HSC engraftment, proliferation and survival [10]. However, it requires complex and expensive aseptic handling procedures (since irradiators are commonly located out of the animal facilities) and specific materials (manipulation, transfer and transportation of animals in sterile conditions) to protect these immunodeficient animals from microbiological agents. The use of such pre-conditioning procedure is thus very stressing for animals. From that point of view, the conditioning of NSG mice by busulfan injections, represents an interesting alternative to irradiation [8]. Moreover, it allows adapting the dose of drug delivered according to each animal weight whereas irradiation (effected in compartmentalized cassettes) could result in slightly different absorbed doses if the individual mice are of very different weight. Based on this approach, we chose to test the effects of different doses of busulfan on NSG mice sensitiveness and engraftment level upon injection of cord blood CD34^+^ cells. We confirmed the data from Robert-Richard et al. [8] obtained with NOD/Scid mice since we showed that 2x25 mg/kg Busulfan provided the optimal huCD45 BM chimerism in mice. However, the fact that busulfan dose did not influence the number of huCFU in recipients’ bone marrow (Figure 1E) suggests that the conditioning could be realized in a relatively large dose-range without affecting the engraftment of more immature SRC. This point is supported by two studies showing that mouse pre-conditioning regimen might not be critical for long-term SRC engraftment even in particular contexts in which the combination of the robustness NOD/SCID IL-2Rgamma-/- (NOG) mouse strain and the sensitivity of intra bone marrow transplantation technique is mandatory [11] or when classical NOD/SCID mice received a multiple-day injection of cells [12]. We also found that males were more sensitive to lower doses of busulfan treatment than females (weight loss (16% vs 10%) and mortality (11% vs 8%)) (Figure 1A to C). In spite of fact that these data are not original they confirmed those from Notta et al. [13] showing that females were more “permissive” than males to engraftment of human cells (Figure 4) whatever the cell number injected (from 100 to 1000 cells). Here, our data suggest that the 100 CD34^+^ cord blood cells dose corresponds to the limiting dilution for NSG males since around 50% of mice were negative for huCD45 whereas more than 95% of females were positive. The lower level of the limiting dilution threshold in females argues for a better sensitivity of female NSG mice to human HSC engraftment. This result is completely in accordance with previous published study in which the very SRC-enriched population “Lin^-^CD34^+^CD38^-^CD90^+^CD45RA^-^“ was injected using the strongly efficient intra femoral route in sub-lethally irradiated NSG mice. In this context, the authors also showed that females were better engrafted than males [13], evidencing thus that this difference between males and females mice in terms of engraftment sensitivity persists whatever the injection route, pre-conditioning technique or SRC enrichment. In addition to the better immunodeficient status of females compared to males, and some sex-associated factors, such as steroid hormones that can positively or negatively regulate human HSCs considered by these authors [13], we propose that the weight of animal could also be involved in this phenomenon. Indeed, even we usually used males and females having the same age, we observed a 16% higher weight of males compare to females (mean weight was 24.4g (n=75) and 20.3g (n=40) for male and female cohorts respectively). In this situation, even if the same number of cells per mice was injected, the cell dose/kg of body weight is in fact, lower in males. This could partly explain the difference in engraftment level. Taken together, our data presented in Figure 1 led us to use female mice conditioned with 2 injections of 25 mg/kg of busulfan in most of our further experiments. Very interestingly, using these conditions, we were able to increase the delay between pre-conditioning and cell injections from 24 hours up to 7 days without significantly altering the huCD45 chimerism, the balance between myeloid and B lymphoid cells and the huCFU number. This observation is of major practical interest because it largely increases the experimental time-schedule flexibility that is frequently requested in human SRC experiments. Intra venous, intra bone marrow and intra peritoneal injections of human cells in NOD/SCID, NOG or NSG mice are described in the literature for SRC studies [14]. Here, we confirmed that the huCD45 chimerism in mice bone marrow is not significantly affected by the type of intra venous injection i.e intra caudal and retro orbital routes (Figure 3) [15,16].

However we noticed that 2 mice out of 16 injected in the caudal vein did not show any human chimerism whereas all the 21 mice injected in the retro orbital sinus exhibited human engraftment. Moreover, the significantly higher number of huCFU detected in the bone marrow of recipients injected in the retro orbital sinus compared to the one in the caudal vein suggests that this route allows a better HSC homing or “nichage”. Despite frequent subjective reluctances of researchers to its use, the retro-orbital injection route is in fact less painful and stressful for animals [16,17] than alternative intravascular injection techniques. Indeed, the technically challenging injection in the caudal vein leads to some failures even for skilled experimentators (2 non engrafted mice in our study). Moreover, this procedure requires the heating of mice (under a lamp or a slightly heating block) to promote peripheral vasodilation that facilitates the vein catheterism and cell injection [17] and it causes distress in the nonanesthetized animals, especially if the initial venipuncture is unsuccessful and repeated attempts are made. Taken together our data lead us to propose that SRC studies have to be performed with female NSG mice, conditioned with 2 IP injections of 25mg/kg of busulfan before CD34+ cells injection in the retro orbital sinus. Very interestingly, we provide the first comparative study which demonstrates that increasing the usual 24h period between conditioning and cell transplantation of NSG mice up to 7 days resulted in similar percentages and types of BM chimerism at 8 weeks post transplantation. Our data could facilitate the development of *in vivo* studies with human CD34^+^ cells by improving the flexibility of experimental protocols without altering the reliability of the results.
